# Adverse effects of daily oral pre-exposure prophylaxis in men who have sex with men and transgender women: a systematic review and meta-analysis

**DOI:** 10.1590/0102-311XEN089522

**Published:** 2023-12-08

**Authors:** Marcos Pereira, Caroline Tianeze de Castro, Laio Magno, Tarcio de Almeida Oliveira, Fabiane Soares Gomes, Fabiane Maria Fernandes Neves, Pedro Rafael dos Santos do Nascimento, Ines Dourado

**Affiliations:** 1 Instituto de Saúde Coletiva, Universidade Federal da Bahia, Salvador, Brasil.; 2 Departamento de Ciências da Vida, Universidade do Estado da Bahia, Salvador, Brasil.; 3 Programa de Pós-graduação em Farmácia, Universidade Federal da Bahia, Salvador, Brasil.

**Keywords:** Pre-Exposure Prophylaxis, Drug-Related Side Effects and Adverse Reactions, HIV, Emtricitabine, Tenofovir Disoproxil Fumarate Drug Combination, Profilaxia Pré-Exposição, Efeitos Colaterais e Reações Adversas Relacionados a Medicamentos, HIV, Combinação Emtricitabina e Fumarato de Tenofovir Desoproxila, Profilaxis Pre-Exposición, Efectos Colaterales y Reacciones Adversas Relacionados con Medicamentos, VIH, Combinación Emtricitabina y Fumarato de Tenofovir Disoproxil

## Abstract

The adverse effects of oral pre-exposure prophylaxis (PrEP) using tenofovir disoproxil fumarate are barriers to PrEP initiation and continuation. Although serious effects are rare and predictable, evidence for this assessment among men who have sex with men (MSM) and transgender women (TGW) is still limited. This study assesses the adverse effects of daily oral PrEP in MSM and TGW. This is a systematic review and meta-analysis of clinical trials and cohort studies on the use of daily oral PrEP selected from the PubMed/MEDLINE, Embase, LILACS, and Cochrane CENTRAL databases. Data extraction included adverse effects and changes in renal and hepatic markers. Random effects models were used to summarize the risk of adverse effects throughout the study. Heterogeneity was assessed using the Cochran’s Q test and the inconsistency test (I^2^). The risk of bias and the certainty of the evidence were assessed using the Cochrane Collaboration recommendations. The search identified 653 references. Of these, 10 were selected. All studies assessed the eligibility of renal and hepatic markers. The use of daily oral PrEP was not associated with grade 3 or 4 adverse events (RR = 0.99; 95%CI: 0.83-1.18; I^2^ = 26.1%), any serious adverse event (RR = 1.04; 95%CI: 0.58-1.87; I^2^ = 88.4%), grade 3+4 creatinine level (RR = 0.66; 95%CI: 0.24-1.84; I^2^ = 79.9%), and grade 3 or 4 hypophosphatemia (RR = 0.56; 95%CI: 0.15-2.10). The certainty of the evidence ranged from high to moderate for the outcomes analyzed. Daily oral PrEP is safe and well tolerated by MSM and TGW. Adverse effects were minimal and evenly distributed between intervention and control.

## Resumo

Os efeitos adversos da profilaxia pré-exposição (PrEP) oral com fumarato de tenofovir desoproxila são barreiras para o início e a continuidade da PrEP. Embora os efeitos graves sejam raros e previsíveis, as evidências dessa avaliação entre homens que fazem sexo com homens (HSH) e mulheres transgênero (MTG) ainda são limitadas. Este estudo avalia os efeitos adversos da PrEP oral diária em HSH e MTG. Trata-se de uma revisão sistemática e metanálise de ensaios clínicos e coortes que demonstram o uso de PrEP oral diária selecionados nas bases de dados PubMed/MEDLINE, Embase, LILACS e Cochrane CENTRAL. A extração de dados incluiu os efeitos adversos e alterações nos marcadores renais e hepáticos. Modelos de efeitos aleatórios foram usados para resumir o risco de efeitos adversos ao longo do estudo. A heterogeneidade foi avaliada pelo teste Q de Cochran e inconsistência (I^2^). O risco de viés e a certeza da evidência foram avaliados por meio das recomendações da Colaboração Cochrane. Foram identificadas 653 referências. Destes, dez foram selecionadas. Todos os estudos avaliaram marcadores renais de elegibilidade e marcadores hepáticos. O uso diário de PrEP oral não foi associado a eventos de grau 3 ou 4 (RR = 0,99; IC95%: 0,83-1,18; I^2^ = 26,1%), a qualquer evento adverso grave (RR = 1,04; IC95%: 0,58-1,87; I^2^ =88,4%), à creatinina grau 3 ou 4 (RR = 0,66; IC95%: 0,24-1,84; I^2^ = 79,9%) e à hipofosfatemia grau 3 ou 4 (RR = 0,56; IC95%: 0,15-2,10). A certeza das evidências variou de alta a moderada para os desfechos analisados. A PrEP oral diária é segura e bem tolerada por HSH e MTG. Os efeitos adversos foram mínimos e distribuídos uniformemente entre a intervenção e o controle.

## Resumen

Los efectos adversos de la profilaxis preexposición (PrEP) oral con fumarato de disoproxilo de tenofovir son barreras para el inicio y la continuación de la PrEP. Aunque los efectos graves son raros y predecibles, la evidencia de esta evaluación entre hombres que tienen sexo con hombres (HSH) y mujeres transgénero (MTG) sigue siendo limitada. Este estudio evalúa los efectos adversos de la PrEP oral diaria en HSH y MTG. Se trata de una revisión sistemática y un metaanálisis de ensayos clínicos y cohortes que demuestran el uso de la PrEP oral diaria seleccionada de las bases de datos PubMed/MEDLINE, Embase, LILACS y Cochrane CENTRAL. La recolección de datos incluyó efectos adversos y cambios en los marcadores renales y hepáticos. Se utilizaron modelos de efectos aleatorios para resumir el riesgo de efectos adversos a lo largo del estudio. La heterogeneidad se evaluó mediante la prueba Q de Cochran y la inconsistencia (I^2^). El riesgo de sesgo y la certeza de la evidencia se evaluaron utilizando las recomendaciones de la Colaboración Cochrane. Se identificaron 653 referencias. De estas, se seleccionaron diez. Todos los estudios evaluaron los marcadores renales de elegibilidad y los marcadores hepáticos. El uso diario de la PrEP oral no se asoció con eventos de grado 3 o 4 (RR = 0,99; IC95%: 0,83-1,18; I^2^ = 26,1%), con ningún evento adverso grave (RR = 1,04; IC95%: 0,58-1,87; I^2^ = 88,4%), con creatinina de grado 3 o 4 (RR = 0,66; IC95%: 0,24-1,84; I^2^ = 79,9%) y con hipofosfatemia de grado 3 o 4 (RR = 0,56, IC95%: 0,15-2,10). La certeza de la evidencia varió de alta a moderada para los resultados analizados. La PrEP oral diaria es segura y bien tolerada por HSH y MTG. Los efectos adversos fueron mínimos y se distribuyeron uniformemente entre la intervención y el control.

## Introduction

Pre-exposure prophylaxis (PrEP) is one of the key combination prevention strategies to control the HIV epidemic, especially for populations at substantial risk of HIV infection ^1^, as recommended by the World Health Organization (WHO) in September 2015. Oral PrEP with tenofovir disoproxil fumarate/emtricitabine (TDF/FTC) is highly effective in preventing HIV infection when used as recommended by WHO guidelines ^2^. The use of PrEP requires an initial clinical assessment by anamnesis, an evaluation of contraindications, and monitoring of adverse events.

Although oral PrEP is well tolerated, it can lead to mild or moderate adverse events and rarely to severe conditions ^3^. Therefore, due to the risk of nephrotoxicity, the WHO recommends that participants undergo medical examinations before starting PrEP, to identify any history of kidney injury and thus exclude the association with the medication after initiation ^4^. Moreover, creatinine levels should be measured during PrEP initiation and every six months, with more frequent monitoring in individuals with kidney-related comorbidities and less frequent monitoring in individuals aged < 45 years ^4^.

Evidence from clinical trials and cohort studies on the use of PrEP shows rare adverse effects and changes in renal ^3^ ^,^ ^5^ ^,^ ^6^ and bone markers ^7^. These changes are generally mild and do not lead to significant effects ^8^. However, these studies analyzed multiple groups, but not specifically men who have sex with men (MSM) or transgender women (TGW). A study with a representative sample of transgender individuals in the United States recorded higher rates of certain adverse effects associated with PrEP, such as nausea, diarrhea, kidney failure, and changes in bone density, compared with studies that included only MSM in the same country, despite the limitations and differences in the assessment of these outcomes between the studies ^9^ ^,^ ^10^.

A systematic review found that the risk of a decline in estimated creatinine clearance may differ slightly according to gender ^5^, but cisgender and transgender or non-binary individuals showed no difference regarding risk, although data were scarce. Therefore, it is essential to understand that these findings are not universally applicable to all individuals in each group. Therefore, investigating the adverse effects of PrEP may increase the knowledge about these effects in PrEP users. Moreover, as adverse events can affect the effectiveness of medications ^11^, estimating the adverse effects associated with PrEP in MSM and TGW is important, since they are at a high risk of HIV infection.

Low adherence to PrEP among individuals for whom it is indicated is a substantial problem affecting many groups. The short- and long-term safety of PrEP among individuals at risk of HIV infection raises doubts. Qualitative studies with MSM and TGW reported that concerns about side effects were associated with a lower willingness to take PrEP ^9^ ^,^ ^12^ ^,^ ^13^ ^,^ ^14^, as well as a lack of research with TGW ^12^. Moreover, individuals from different social groups, such as MSM and TGW, may have different risks of adverse reactions to medications, which may be related to failures in treatment follow-up due to factors that alter the risk of problem occurrence or monitoring ^9^ ^,^ ^12^ ^,^ ^14^ ^,^ ^15^. Notably, these groups are a priority for HIV prevention, especially transgender individuals considering using PrEP to prevent HIV, who are concerned about the adverse effects and the interactions of the medication with gender-affirming hormone therapy ^9^ ^,^ ^15^.

Therefore, assessing the adverse effects of using oral PrEP in different key populations, such as MSM and TGW, can provide a better understanding of adverse effects in these populations, since systematic reviews have not yet stratified the adverse effects of subgroups ^3^ ^,^ ^6^, focusing, when available, on renal parameters ^5^. On the other hand, fear of the adverse effects of PrEP is considered a barrier for individuals start using PrEP ^16^ ^,^ ^17^.

Understanding the barriers to PrEP use and producing new evidence on the topic, such as the side effects of PrEP, is essential to ensure its effective implementation ^18^, particularly among populations with disproportionate and/or increasing rates of HIV infection. Moreover, the use of oral PrEP has increased, along with the evaluation of recommendations for monitoring its adverse effects. Therefore, this study aimed to assess the adverse effects of daily oral PrEP in MSM and TGW.

## Methods

### Protocol and registration

This systematic review was conducted according to the *Preferred Reporting Items for Systematic Reviews and Meta-Analyses* (PRISMA) guidelines ^19^ and was based on the methodological recommendations of the Cochrane Collaboration ^20^. The study protocol was registered in the PROSPERO database (protocol n. CRD42020203079).

The study answers the research question: “What are the adverse effects of oral PrEP in MSM and TGW compared with individuals who do not use this prophylaxis?”.

### Eligibility criteria

The PICOT structure was used to define the following eligibility criteria:

The populations of interest (P) were MSM and TGW at any age, regardless of sexual orientation;

The intervention (I) considered was the daily use of oral PrEP: (i) emtricitabine 200mg + tenofovir disoproxil fumarate 300mg (FTC/TDF); (ii) emtricitabine 200mg + tenofovir alafenamide (TAF) 25mg (FTC/TAF); or (iii) tenofovir disoproxil fumarate (TDF);

The comparison group (C) consisted of individuals who did not use PrEP (control group). A comparison group was included to avoid or control for possible nocebo effects in adverse events between the groups;

The outcomes of interest (O) were any serious adverse event, any grade 3 or 4 event, total grade creatinine, and grade 3 or 4 hypophosphatemia (Material Suplementar 1: https://cadernos.ensp.fiocruz.br/static//arquivo/suppl-1-e00089522_4764.pdf)

The study design (T) included cohort studies and clinical trials on PrEP.

This study did not apply restrictions on age, origin, or language of publication. Studies on women, mixed groups (e.g., female sex workers and MSM), serodiscordant heterosexual couples, and sex workers were excluded.

### Search strategy

Searches were performed in the bibliographic databases PubMed/MEDLINE, Embase, Cochrane Central Register of Controlled Trials, LILACS, and OpenGray in May 2020 and updated in April 2022. Medical Subject Headings (MeSH), Emtree, and Health Sciences (DeCS) keywords were used to identify studies published in these databases: “Pre-Exposure Prophylaxis”, “chemoprevention”, “HIV”, “human immunodeficiency virus infection”, “Drug-Related Side Effects and Adverse Reactions”, and “Adverse Drug Reaction”. These keywords were combined with the Boolean operators “OR” and “AND” and their entry terms in all databases. This study also searched grey literature in ProQuest and references to systematic reviews on PrEP to identify studies not included in the electronic search. Material Suplementar 2(https://cadernos.ensp.fiocruz.br/static//arquivo/suppl-2-e00089522_8074.pdf) shows the details of the search process.

### Study selection

The publications found in the databases were inserted into the Rayyan application (https://www.rayyan.ai/), a free software that helps select studies. Two evaluators (M.P. and T.A.O.) independently screened titles and abstracts to identify potentially eligible studies. The eligibility of the publications that met the inclusion criteria in the initial phase was confirmed by reading them in full. Studies that met all eligibility criteria were included in the qualitative synthesis. Disagreements regarding the inclusion of studies were resolved by a third evaluator (L.M.).

### Data extraction

Using a standardized form, the reviewers (M.P., C.T.C., T.A.O., F.S.G., P.R.S.N., and F.M.F.N.) independently extracted data from the included studies. Extracted data included year of publication, study design, study site, sample size, mean age of participants, medications used, adverse reactions identified, and the criteria and frequency of measurement of adverse effects. At the end of this study, the lead author (M.P.) reviewed all the information. Moreover, authors whose studies were not available in the databases were contacted by the corresponding author to request the full text.

### Methodological quality assessment

The methodological quality of all the studies that met the eligibility criteria was assessed using the risk of bias scale for estimates of effectiveness and safety in non-randomized intervention studies recommended by the Cochrane Collaboration, the *Risk of Bias in Non-randomized Studies of Interventions* (ROBINS-I) ^21^. This tool assesses seven domains of bias classified by moment of occurrence: pre-intervention (bias due to confounding and bias in selection of participants into the study), at intervention (bias in classification of interventions), and post-intervention (bias due to deviations from intended interventions, bias due to missing data, bias in measurement of outcomes, and bias in selection of the reported result).

The items were classified as low, moderate, severe, or critical risk of bias or no information, according to the descriptions in the *Cochrane Handbook for Systematic Reviews of Interventions* ^20^.

### Statistical analysis

A random effects meta-analysis was conducted using the rate of adverse events for the following outcomes: any serious adverse event, any grade 3 or 4 event, total grade creatinine (subgroups 1+2 and 3+4), and grade 3 or 4 hypophosphatemia (Material Suplementar 1: https://cadernos.ensp.fiocruz.br/static//arquivo/suppl-1-e00089522_4764.pdf). These are described as follows:

Any serious adverse event: any unforeseen medical event that, at any dose, leads to death, is life-threatening, requires hospitalization or prolongation of an existing hospitalization, or causes persistent or significant disability or incapacity ^22^;

Any grade 3 or 4 event: severe or potentially life-threatening event;

Total grade creatinine (subgroups 1+2 and 3+4): all serum creatinine elevations from 1.1 to 1.3 times the upper limit of typical levels. Grade 2 and higher events include serum creatinine elevations of 1.3 to 1.8 times the upper limit of typical levels or 1.3 to 1.5 times the participants’ baseline value ^22^;

Hypophosphatemia: grade 3 includes serum phosphate < 2.0-1.0mg/dL or < 0.6-0.3mmol/L. Grade 4 includes serum phosphate < 1.0mg/dL or < 0.3mmol/L and life-threatening consequences ^22^.

These biochemical outcomes were selected because when they are altered in individuals under PrEP, discontinuation is recommended. In studies with no events in the intervention or control groups, a value of one was entered to estimate the summary mean.

The measures adopted to summarize the results were the relative risk (RR) and their respective 95% confidence intervals (95%CI). Cochran’s Q statistical test and the inconsistency test (I^2^) were used to assess the heterogeneity and consistency of the studies ^23^. In the presence of heterogeneity (p < 0.05; I^2^ > 25%), a random model with inverse variance was used, weighted by the results of the individual studies ^24^. A minimum of eight studies were considered to assess publication bias by preparing the funnel plot and performing Egger’s test ^20^ ^,^ ^25^.

### Assessment of the certainty of the evidence

The certainty of the evidence was assessed using GRADEpro software (https://www.gradepro.org/). The GRADE (*Grading of Recommendations Assessment, Development, and Evaluation*) system classifies the quality of the evidence into four levels: high, moderate, low, and very low, according to study design limitations, indirect evidence, inconsistency of results, imprecision of results, and a significant probability of publication bias ^26^.

## Results

### Study selection

**Figure 1:**
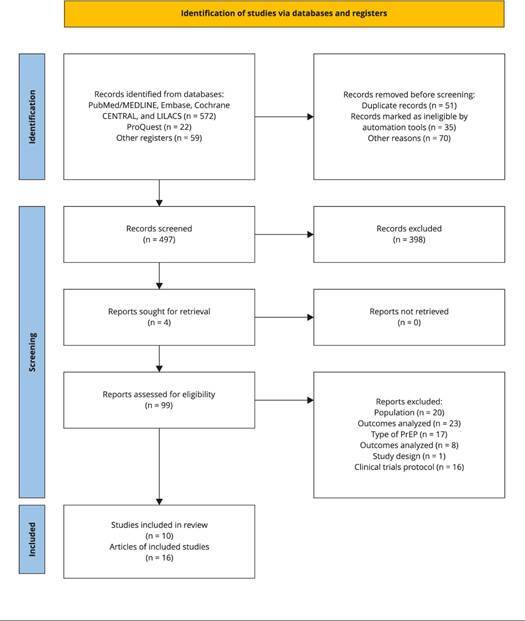
Flowchart of study selection.

We identified 653 references using the search strategies adopted, of which 99 were selected to assess their eligibility and 10 studies, that is, 16 articles ^10^ ^,^ ^27^ ^,^ ^28^ ^,^ ^29^ ^,^ ^30^ ^,^ ^31^ ^,^ ^32^ ^,^ ^33^ ^,^ ^34^ ^,^ ^35^ ^,^ ^36^ ^,^ ^37^ ^,^ ^38^ ^,^ ^39^ ^,^ ^40^ ^,^ ^41^ were included in the systematic review (Figure 1). The exclusion criteria involved the study population (n = 20), the outcomes analyzed (n = 23), the PrEP regimen (n = 17), the clinical trial protocol (n = 15), and the cross-sectional study design (n = 1) (Figure 1).

PrEP: pre-exposure prophylaxis. Note: the reasons for excluding publications can be accessed in the Supplementary Material 3 (https://cadernos.ensp.fiocruz.br/static//arquivo/suppl-3-e00089522_3123.pdf).

### Assessment of risk of bias

**Figure 2:**
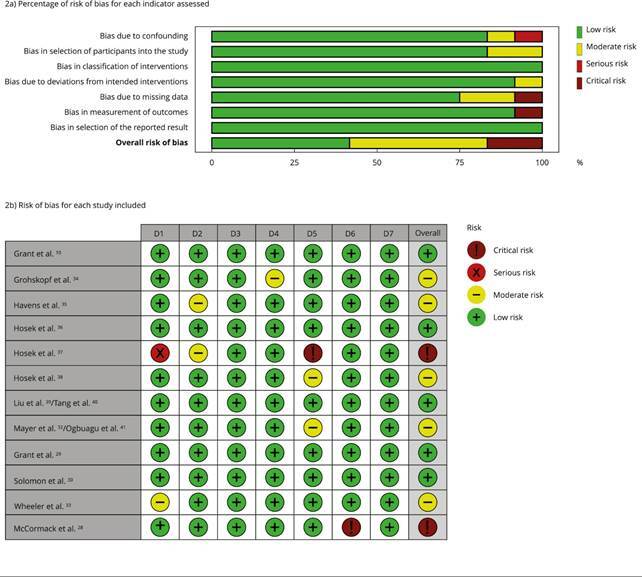
Assessment of risk of bias in non-randomized studies (ROBINS-I - *Risk of Bias in Non-randomized Studies of Interventions*).

Figure 2 shows the results of the quality assessment of the included studies. Among them, risk of bias was predominantly low ^10^ ^,^ ^29^ ^,^ ^30^ ^,^ ^36^ ^,^ ^39^ ^,^ ^40^ and moderate ^32^ ^,^ ^33^ ^,^ ^34^ ^,^ ^38^ ^,^ ^41^, and two were critical ^28^ ^,^ ^37^. The main criteria contributing to moderate or critical risk of bias were bias due to confounding, bias due to missing data, and bias in in measurement of outcomes.

Domains: D1 - bias due to confounding; D2 - bias in selection of participants into the study; D3 - bias in classification of interventions; D4 - bias due to deviations from intended interventions; D5 - bias due to missing data; D6 - bias in measurement of outcomes; D7 - bias in selection of the reported result.

### Characterization and qualitative synthesis of the selected studies

Table 1 presents the main characteristics of the included studies. We analyzed information on adverse effects (US CDC PrEP ^31^ ^,^ ^34^, iPrEx ^10^ ^,^ ^27^ ^,^ ^30^, US PrEP Demonstration Project ^39^ ^,^ ^40^, and PrEPare ^36^ ^,^ ^37^) and study extension (PrePare ATN 08 3MV ^38^, ATN 117 ^35^, ADAPT Study ^29^, HPTN 073 ^33^, DISCOVER ^32^ ^,^ ^41^, and PROUD ^28^.

**Table 1:** Characteristics of selected studies on adverse effects of daily oral pre-exposure prophylaxis (PrEP) in men who have sex with men (MSM) and transgender women (TGW).

Authors (Year)	Study	Study design	Medication	Population	Age (years)	Countries	Follow-up	Participants	Hormone use in the PrEP group
Liu et al. ^31^ (2011)	US CDC PrEP	Phase II randomized, double-blind, placebo-controlled extended safety trial	Daily oral TDF 300mg	MSM	18-60	United States	24 months (immediate arm) or 15 months (delayed arm)	184	Testosterone and growth hormone (17%)
Grohskopf et al. ^34^ (2013)	US CDC PrEP	Randomized, double-blind, placebo-controlled trial	Daily oral TDF 300mg	MSM	18-60	United States	24 months	400	Not reported
Grant et al. ^10^ (2010)	IprEx	Phase III randomized, double-blind, placebo-controlled study	TDF/Daily FTC	MSM/TGW	18-67	Brazil, Ecuador, Peru, South Africa, Thailand, and the United States	1.2-2.8 years	2,499	Not reported
Solomon et al. ^30^ (2014)	IprEx	Phase III randomized, double-blind, placebo-controlled study	TDF/Daily FTC	MSM/TGW	18-67	Brazil, Ecuador, Peru, South Africa, Thailand, and the United States	1.2-2.8 years	2,499	Not reported
Deutsch et al. ^27^ (2015)	IprEx	Phase III randomized, double-blind, placebo-controlled study	TDF/Daily FTC	MSM/TGW	18-67	Brazil, Ecuador, Peru, South Africa, Thailand, and the United States	2 years	2,499	Exogenous female hormone (20%)
Liu et al. ^39^ (2016)	US PrEP Demonstration Project	Prospective cohort study	TDF/Daily FTC	MSM/TGW	18-65	United States	48 weeks	557	Testosterone or anabolic steroid (1.5%)
Tang et al. ^40^ (2018)	US PrEP Demonstration Project	Prospective cohort study	TDF/Daily FTC	MSM/TGW	18-65	United States	48 weeks	557	Not reported
McCormack et al. ^28^ (2016)	PROUD	Open-label randomized trial	TDF/Daily FTC	MSM	29-43	United Kingdom	48 weeks	544	Not reported
Hosek et al. ^38^ (2013)	PrEPare ATN 08 3MV	Pilot study using a randomized 3-arm design	TDF/Daily FTC	Young MSM	18-22	United States	24 weeks	68	Not reported
Hosek et al. ^36^ (2017)	PrEPare	Prospective cohort study/PrEP Demonstration Project	TDF/Daily FTC	Young MSM	15-17	United States	48 weeks	78	Not reported
Hosek et al. ^37^ (2017)	PrEPare	Prospective cohort study/PrEP Demonstration Project	TDF/Daily FTC	Young MSM	18-22	United States	48 weeks	200	Not reported
Havens et al. ^35^ (2017)	ATN 117	Prospective *cohort*study/PrEP Demonstration Project	TDF/Daily FTC	Young MSM	15-22	United States	48 weeks	101	Not reported
Wheeler et al. ^33^ (2019)	HPTN 073	Non-randomized open-label PrEP	TDF/Daily FTC	MSM	26 (IQR: 23-32)	United States	52 weeks	161	Anabolic steroids and female sex hormones
Grant et al. ^29^ (2018)	067/ADAPT Study	Phase II randomized, open-label	FTC/Daily vs. non-daily oral TDF	MSM/TGW	≥ 18	Thailand	34 weeks	357	
Mayer et al. ^32^ (2020)	DISCOVER	Phase III randomized, double-blind, multicenter, active-controlled trial	Daily tablets of FTC (200mg) and TAF (25mg)	MSM/TGW	34 (IQR: 28-44)		96 weeks	5,387	Gender-affirming hormone therapy (17 TGW)
Ogbuagu et al. ^41^ (2021)	DISCOVER	Phase III randomized, double-blind, multicenter, active-controlled trial	Daily tablets of FTC (200mg) and TAF (25mg)	MSM/TGW	34 (IQR: 28-44)		96 weeks	5,387	Not reported

FTC: emtricitabine; IQR: interquartile range; TAF: tenofovir alafenamide; TDF: tenofovir disoproxil fumarate.

The studies consisted of clinical trials (n = 7) and cohort studies (n = 3) and mostly involved MSM (n = 4), young MSM (n = 4), and MSM/TGW (n = 2). Moreover, the years of publication ranged from 2011 to 2021, the samples from 78 to 5,387 participants, and the ages from 15 to 67 years. Four studies had a follow-up duration of ≤ 1 year ^28^ ^,^ ^29^ ^,^ ^38^ ^,^ ^40^and the others < 1 year.

Table 2 presents the adverse event monitoring data evaluated at baseline and in the study segment. All studies addressed renal function markers at eligibility or baseline. Hepatic markers were reported in only five studies (US CDC PrEP, iPrEx, PrEPare, and ADAPT Study). Markers of adverse effects were assessed at different times in the participants’ segment and, in most cases, classified according to the U.S. Division of AIDS criteria ^22^.

**Table 2:** Monitoring of the adverse effects of daily oral pre-exposure prophylaxis (PrEP) in men who have sex with men (MSM) and transgender women (TGW).

Authors (Year)	Study	Study period	Renal function	Hepatic function	Biochemical parameters in eligibility	Monitoring of adverse events	Outcome measures
Liu et al. ^31^ (2011)	US CDC PrEP	February 2005 to July 2007/January 2005 to July 2007	Yes	Yes	Cockroft-Gault creatinine clearance; spot urine calcium/creatinine ratio	Each quarterly visit	None
Grohskopf et al. ^34^ (2013)	US CDC PrEP	February 2005 to July 2007/January 2005 to July 2007	Yes	Yes	Cockcroft-Gault creatinine clearance; serum creatinine; phosphorus	Weeks 1, 3, 6, 9, 12, 15, 18, 21, and 24	DAIDS toxicity tables (January 2004)
Grant et al. ^10^ (2010)	iPrEx	July 2007 to December 2009	Yes	Yes	Serum creatinine; Cockcroft-Gault creatinine clearance; urine dipstick testing for protein and glucose; leukocyte esterase testing; urine phosphorus, calcium, creatinine, uric acid, protein, and glucose	Weeks 4, 8, 12, 16, and 24 and then every 12 weeks	Grade 1 or higher creatinine toxicity; grade 3 or higher phosphorous toxicity; grade 2, 3, or 4 laboratory; DAIDS Table for Grading the Severity of Adult and Pediatric Adverse Events (2004)
Solomon et al. ^30^ (2014)	iPrEx	July 2007 to December 2009	Yes	Yes	Serum creatinine; Cockcroft-Gault creatinine clearance; urine dipstick testing for protein and glucose; leukocyte esterase testing; urine phosphorus, calcium, creatinine, uric acid, protein, and glucose	Weeks 4, 8, 12, 16, and 24 and then every 12 weeks	Grade 1 or higher creatinine toxicity; grade 3 or higher phosphorous toxicity; grade 2, 3, or 4 laboratory; DAIDS Table for Grading the Severity of Adult and Pediatric Adverse Events (2004)
Deutsch et al. ^27^ (2015)	iPrEx	July 2007 to December 2009	Yes	Yes	Serum creatinine; Cockcroft-Gault creatinine clearance; urine dipstick testing for protein and glucose; leukocyte esterase testing; urine phosphorus, calcium, creatinine, uric acid, protein, and glucose	Weeks 4, 8, 12, 16, and 24 and then every 12 weeks	Grade 1 or higher creatinine toxicity; grade 3 or higher phosphorous toxicity; grade 2, 3, or 4 laboratory; DAIDS Table for Grading the Severity of Adult and Pediatric Adverse Events (2004)
Liu et al. ^39^ (2016)	US PrEP Demonstration Project	October 1, 2012, to January 23,2014	Yes		Cockcroft-Gault creatinine clearance and eGFR (CKD-EPI); urine protein dipstick test	Weeks 4, 12, 24, 36, and 48	DAIDS adverse event grading table version 1.0, December 2004, and the DAIDS Male Genital Grading Table
Tang et al. ^40^ (2018)	US PrEP Demonstration Project	October 1, 2012, to January 23,2014	Yes		Cockcroft-Gault creatinine clearance and eGFR (CKD-EPI) Urine protein dipstick test	Weeks 4, 12, 24, 36, and 48	DAIDS adverse event grading table version 1.0, December 2004, and the DAIDS Male Genital Grading Table
McCormack et al. ^28^ (2016)	PROUD	November 2012 to October 2016	Yes		Serum creatinine; urine protein dipstick test	Annualy and every 3 months	None
Hosek et al. ^38^ (2013)	PrEPare ATN 08 3MV	August 2005 to November 2006	Yes	Yes	Hepatic and pancreatic function tests; urine dipstick testing for protein and glucose	Every 4 weeks for 24 weeks	Expedited Adverse Event Reporting (grade 2 and higher)
Hosek et al. ^36^ (2017)	PrEPare	January to September 2013	Yes	Yes	Renal function: phosphate, blood urea nitrogen, creatinine, and urine dipstick testing for protein and glucose; pancreatic function: amylase; hepatic function: AST, ALT, alcaline phosphatase, total bilirubin, and direct bilirubin	Monthly in the first quarter (weeks 4, 8, and 12) and then quarterly until 48 weeks	ATN adverse event severity grading table for adolescents (October 2006 to March 2011)/Manual for Expedited Reporting of Adverse Events to DAIDS (version 2.0, March 2011)
Hosek et al. ^37^ (2017)	PrEPare	August 2013 to September 2014	Yes	Yes	Renal function: phosphate, blood urea nitrogen, creatinine, and urine dipstick testing for protein and glucose; pancreatic function: amylase; hepatic function: AST, ALT, alcaline phosphatase, total bilirubin, and direct bilirubin	Monthly in the first quarter (weeks 4, 8, and 12) and then quarterly until 48 weeks	ATN adverse event severity grading table for adolescents (October 2006 to March 2011)/Manual for Expedited Reporting of Adverse Events to DAIDS (version 2.0, March 2011)
Havens et al. ^35^ (2017)	ATN 117	December 2012 to October 2014	Yes	Yes	Serum creatinine, albumin, calcium, phosphate, glucose, protein, and retinol binding protein	Weeks 4, 8, 12, 24, 36, and 48	None
Grant et al. ^29^ (2018)	067/ADAPT Study	July 4, 2012, to May 6, 2014	Yes	Yes	Renal function: estimated creatinine clearance, phosphate; hepatic function: AST and ALT	Weeks 4, 10, 18, and 30	None
Wheeler et al. ^33^ (2019)	HPTN 073	February 2013 to September 2014	Yes		Cockcroft-Gault creatinine clearance; urine dipstick testing for protein and glucose	At screening, 4 and 13 weeks after inclusion, and then quarterly; at screening and quarterly after inclusion	None
Mayer et al. ^32^ (2020)	DISCOVER	September 13, 2016, to June 30, 2017	Yers		Cockcroft-Gault creatinine clearance; urinary RBP; lipids and fasting glucose; urine protein and urine protein to creatinine ratio	Weeks 4 and 12 and then every 12 weeks	None
Ogbuagu et al. ^41^ (2021)	DISCOVER	September 13, 2016, to June 30, 2017	Yes		Cockcroft-Gault creatinine clearance ; urinary RBP; lipids and fasting glucose; urine protein and urine protein to creatinine ratio	Weeks 4 and 12 and then every 12 weeks	None

ALT: alanine aminotransferase; AST: aspartate aminotransferase; ATN: Adolescent Trials Network; DAIDS: U.S. Division of AIDS; eGFR: estimated glomerular filtration rate; RBP: retinol binding protein.

### Meta-analysis results

**Figure 3:**
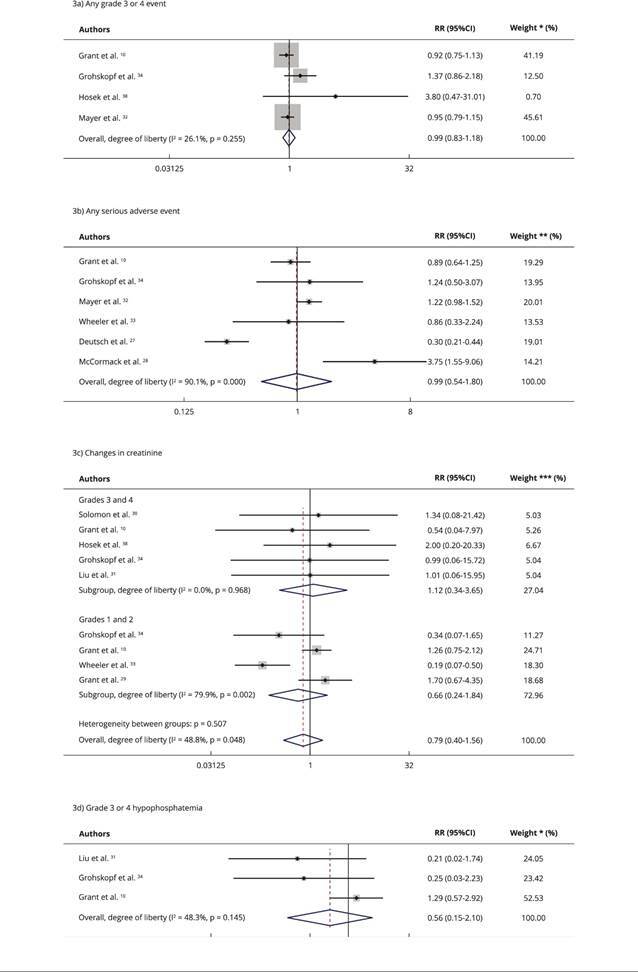
Forest plots for adverse effects of the use of daily oral pre-exposure prophylaxis (PrEP) in men who have sex with men (MSM) and transgender women (TGW).

Figure 3 shows the results of the meta-analysis. Publication bias could not be assessed due to the small number of studies analyzed. We obtained the following results:

95%CI: 95% confidence interval; RR: relative risk. * The weights are from the random effects model; ** The weights are from the random effects model; continuity correction applied to studies with zero cells; *** The weights and the test for heterogeneity between subgroups are from the random effects model.

Any grade 3 or 4 event: no statistically significant effect (RR = 0.99; 95%CI: 0.83-1.18; I^2^ = 26.1%) on the total number of grade 3 or 4 adverse events in PrEP users compared with the control group (Figure 3a);

Any serious adverse event: six studies reported serious adverse effects. The use of oral PrEP was not associated with serious adverse effects (RR = 0.99; 95%CI: 0.54-1.80; I^2^ = 90.1%) (Figure 3b);

Creatinine changes: four studies reported data on serious adverse events related to creatinine, but only two were included in the meta-analysis due to the number of observations (> 0). The use of daily oral PrEP was not associated with the occurrence of grade 1 or 2 (RR = 1.12; 95%CI: 0.34-3.65; I^2^ = 0%) or grade 3 or 4 creatinine levels (RR = 0.66; 95%CI: 0.24-1.84; I^2^ = 79.9%) (Figure 3c);

Grade 3 or 4 hypophosphatemia: the meta-analyses of grade 3 or 4 hypophosphatemia found no significant difference (p = 0.53) between the number of events in PrEP users compared with the control group (RR = 0.56; 95%CI: 0.15-2.10). Heterogeneity between trials was moderate (I^2^ = 48.3%) (Figure 3d).

### Certainty of evidence

The certainty of the evidence for any grade 3 or 4 event and creatinine changes was high. However, it was moderate for the outcomes of any serious adverse event and grade 3 or 4 hypophosphatemia due to the high unexplained heterogeneity, the few included studies in the meta-analysis, and the number of outcome observations (Table 3).

**Table 3:** Certainty of the evidence of the outcomes included in the meta-analysis. Adverse effects of oral daily pre-exposure prophylaxis (PrEP) with tenofovir disoproxil fumarate (TDF) in men who have sex with men (MSM) and transgender women (TGW).

Outcome	Participants (studies)	Relative effect [RR (95%CI)]	Anticipated absolute effects [% (95%CI)]			Certainty *	Outcome
			Without PrEP	With PrEP	Difference		
Any grade 3 or 4 event	7,541 (4 RCTs)	0.99 (0.83-1.18)	10.6	10.4 (8.8-12.5)	0.1% lower (1.8 lower to 1.9 higher)	⨁⨁⨁⨁ High	Daily oral PrEP use was not associated with any grade 3 or 4 event
Any serious adverse event	8,683 (7 RCTs)	1.04 (0.58-1.87)	5.9	6.2 (3.4-11.1)	0.2% higher (2.5 lower to 5.2 higher)	⨁⨁⨁◯ Moderate **	Daily oral PrEP use was not associated with any serious adverse event
Changes in creatinine	4,946 (6 RCTs)	0.79 (0.40-1.56)	1.9	1.5 (0.8-3.0)	0.4% lower (1.2 lower to 1.1 higher)	⨁⨁⨁⨁ High	Daily oral PrEP use was not associated with renal dysfunction
Grade 3 or 4 hypophosphatemia	3,145 (3 RCTs)	0.56 (0.15-2.10)	1.2	0.7 (0.2-2.5)	0.5% lower (1.0 lower to 1.3 higher)	⨁⨁⨁◯ Moderate ***	Daily oral PrEP use was not associated with renal dysfunction

95%CI: 95% confidence interval; RCT: randomized clinical trial; RR: risk ratio.

Note: the risk in the intervention group (and its 95%CI) is based on the assumed risk in the comparison group and the relative effect of the intervention (and its 95%CI).

* GRADE levels of evidence: High certainty (the authors have a lot of confidence that the true effect is similar to the estimated effect), Moderate certainty (the authors believe that the true effect is probably close to the estimated effect, but could also be markedly different), Low certainty (the true effect might be markedly different from the estimated effect), and Very low certainty (the true effect is probably markedly different from the estimated effect);

** High unexplained heterogeneity;

*** Few studies included in the meta-analysis with a small number of outcome observations.

## Discussion

### Main findings of the review

In this study, we reviewed clinical trials and cohort studies on the adverse effects of oral PrEP in MSM and TGW. This is the first systematic review to assess the adverse effects of daily oral PrEP in MSM and TGW.

The results of this systematic review showed that most studies on PrEP use did not present a risk of serious adverse events. The meta-analysis confirmed these observations, with the lack of statistically significant association with serious adverse outcomes in the control group. Daily oral PrEP in MSM, young MSM, and TGW showed no statistically significant association with the occurrence of serious adverse events, grade 3 or 4 adverse events, serious changes in creatinine levels (grade 3+4), and grade 3 or 4 hypophosphatemia. Thus, this study showed that the daily use of PrEP was safe and well tolerated in the study population.

These findings are important, since adverse events can reduce adherence to PrEP. Adherence is a major challenge for effective PrEP implementation, particularly among young MSM and TGW ^16^ ^,^ ^42^. Although our meta-analysis focused on clinical trials and cohort studies, the adverse events found in the studies may determine the effectiveness of this therapy in preventing HIV in MSM and TGW. Moreover, our findings may help prescribers assess the risk-benefit of the use of PrEP by MSM and TGW in clinical practice.

Most studies assessed baseline renal parameters and the eligibility of participants. However, these studies did not address renal markers ^28^. Renal function was assessed predominantly by measuring serum creatinine levels and estimating creatinine clearance using the Cockcroft-Gault equation ^43^. Another correlation of renal function assessment analyzed in this study was the serum phosphate level measured in PrEP users in three studies. Only four studies assessed hepatic function, considering liver transaminases (aspartate aminotransferase and alanine aminotransferase) as the main factors.

In the segment of PrEP users, studies have no consensus on the evaluation period of the analyzed renal and hepatic markers. However, many of these studies reported consistent associations, showing that daily oral PrEP does not pose a substantial risk of serious adverse events.

These results are greatly relevant, especially for TGW, since their hormone therapy is often based on a combination of estradiol and an antiandrogen ^44^. The use of these substances along with PrEP could potentiate or lead to serious adverse reactions, which were not observed in this meta-analysis.

### Comparisons with other studies in the literature

The results of this study, particularly the association between the use of TDF/FTC and the risk of adverse events, are in line with other studies. A systematic review and meta-analysis of 13 studies that compared 15,678 randomized participants who used PrEP (TDF/FTC or TDF) with individuals who used a placebo or received no treatment found no significant difference in the risk of grade 3 or 4 clinical adverse events or serious adverse effects between the groups. Moreover, the authors found no significant difference in the risk of specific adverse renal or bone outcomes ^3^.

A meta-analysis evaluating the effect of PrEP on serum creatinine level using 10 clinical trials that included 17,220 participants randomized to daily oral PrEP (n = 9,913) and placebo (n = 7,307) groups found the opposite result ^6^. Participants assigned to the daily PrEP group had a modestly increased risk of grade 1 or higher creatinine events (odds ratio - OR = 1.36; 95%CI: 1.09-1.71). The absolute risk increase was lower (pooled risk increase 0.6%; 95%CI: 0.1-1.2) ^6^. However, these studies were not methodologically adequate to provide robust evidence of this relationship, due to the lack of a subgroup analysis similar to that performed in this study, as well as the risk of bias and the certainty of the meta-analysis evidence.

A systematic review and meta-analysis of individual data from PrEP users also showed results similar to ours regarding serious adverse effects. A meta-analysis of 11 clinical trials with 13,523 participants showed that the use of PrEP increases the risk of grade 1 or higher renal adverse events and grade 2 or higher renal events. However, the association between grade 2 and higher events was not statistically significant. Events are rare, non-progressive, and disappear when PrEP is discontinued ^5^. A subgroup analysis showed that the highest risks were associated with increasing age and baseline creatinine clearance of 60.00-89.99mL/minute. The study highlighted the importance of screening and monitoring renal function in older individuals, individuals with baseline creatinine clearance < 90mL/minute, and individuals with kidney-related comorbidities ^5^. A similar result was identified by the U.S. Preventive Services Task Force review for renal adverse events, but most of them were mild and reversible ^45^.

A recent meta-analysis found that PrEP was safe for MSM, serodiscordant couples, heterosexual individuals, and injecting drug users. However, unrecognized HIV at the time of notification increases the risk of drug-resistant viral mutations ^46^. The meta-analysis of placebo-controlled trial showed no significant difference between the groups for any reported adverse events (RR = 1.01; 95%CI: 0.99-1.03; I^2^ = 42%) and the risk of serious adverse events (RR = 0.91; 95%CI: 0.74-1.13; I^2^ = 67), but the meta-analysis for renal function was not presented ^46^.

These findings reinforce the safety of PrEP, especially in this study with MSM and TGW, who suffered from interpersonal violence, discrimination, and health disparities ^47^, which can alter the risk of occurrence and monitoring of adverse events.

### Limitations and strengths

This study has some limitations. The studies reviewed showed a high degree of heterogeneity between them. Besides statistical heterogeneity, the studies had different designs and involved different dosages, duration of exposure, follow-up time, time to event, and frequency of assessment of adverse effects, which may influence our results.

Some studies did not report the criteria for assessing adverse effects according to the U.S. Division of AIDS ^22^, which limited the inclusion of this information in the meta-analysis. These criteria are important, as they consider the evolution of events in the study population. Moreover, most studies were conducted in countries such as the United States, allowing the comparison of results between countries.

This review focused on the assessment of biochemical parameters and did not analyze the adverse effects on bone health markers. We did not assess long-term safety because the maximum follow-up period was two years, focusing only on the use of daily oral PrEP to avoid comparing adverse effects with other types of PrEP. Moreover, the searches were performed by a specialist in systematic reviews and underwent slight variations according to the databases in order to retrieve studies on the topic with more sensitivity. Finally, we did not perform a meta-analysis by type of PrEP, given the small number of studies included in this review and the population, since some studies did not stratify the results for the populations analyzed (MSM and TGW). We also did not perform a subgroup analysis, probably due to the low frequency of adverse events associated with daily oral PrEP.

Despite the limitations imposed by the analyzed studies, they were conducted following rigorous methods, qualifying the findings presented in this review. It is possible to highlight the strengths of this study as a comprehensive review and an extensive search of the scientific literature on the topic, in accordance with the PRISMA and Cochrane Collaboration guidelines. The strengths of this study include the assessment of the risk of bias and the certainty of the evidence. Moreover, the study was methodologically rigorous and was performed by independent reviewers, including a gray literature database.

Another strength of this systematic review and meta-analysis was the focus, except for renal events, on studies with grade 3 or higher events, which are the most dangerous adverse events and may require medical intervention. Finally, this is the first meta-analysis to assess the potential adverse effects of the use of PrEP among MSM and TGW.

### Implications and recommendations

The high and moderate evidence from this review suggests that the use of daily oral PrEP has few adverse effects in MSM and TGW. The total number of adverse events, any grade 3 and 4 adverse effect, and changes in creatinine and phosphate level were similarly distributed between participants using PrEP and the control group. We recommend recording and reporting adverse events in studies that follow the monitoring recommendations ^22^ and increasing the number of studies on the use of PrEP in low- and middle-income countries, since most studies have focused on high and middle-income countries. Health services and policies on PrEP should expand information on the minimal risk and safety of its use by individuals with clinically healthy renal and hepatic function to reduce barriers to PrEP in individuals at increased risk of HIV infection.
