# Supplementary material for: Adverse effects of daily oral pre-exposure prophylaxis in men who have sex with men and transgender women: a systematic review and meta-analysis
Source: Cad Saude Publica. 2023 Dec 8;39(Suppl 1):e00089522. doi: 10.1590/0102-311XEN089522 (PMC10712916; doi:10.1590/0102-311XEN089522)
Supplement: Supplementary file 1 [file 1678-4464-csp-39-s1-EN089522-s1.pdf]

Supplementary Material 1 Creatinine and hypophosphatemia adverse events as defined by the National Institutes of Health, Division of AIDS.

**Parameter**	**Grade 1 (mild)**	**Grade 2 (moderate)**	**Grade 3 (severe)**	**Grade 4 (potentially life-threatening)**
Creatinine	1.1 to 1.3 x ULN	> 1.3 to 1.8 x ULN OR Increase to 1.3 to < 1.5 x participant's baseline	> 1.8 to < 3.5 x ULN OR Increase to 1.5 to < 2.0 x participant's baseline	≥ 3.5 x ULN OR Increase of ≥ 2.0 x participant's baseline
Hypophosphatemia	2.0 to < LLN 0.65 to < LLN	1.4 to < 2.0 0.45 to < 0.65	1.0 to < 1.4 0.32 to < 0.45	< 1.0 < 0.32
