# Supplementary material for: Adverse effects of daily oral pre-exposure prophylaxis in men who have sex with men and transgender women: a systematic review and meta-analysis
Source: Cad Saude Publica. 2023 Dec 8;39(Suppl 1):e00089522. doi: 10.1590/0102-311XEN089522 (PMC10712916; doi:10.1590/0102-311XEN089522)
Supplement: Supplementary file 2 [file 1678-4464-csp-39-s1-EN089522-s2.pdf]

Supplementary Material 2 Search strategies in electronic databases, updated on April 11, 2022.

Database	Search	Strategies	Result
Pubmed/Medline	#1	("Pre-Exposure Prophylaxis"[Mesh]) OR (Pre Exposure Prophylaxis) OR (Pre-Exposure Prophylaxi) OR (Prophylax*, Pre-Exposure) OR (Pre-Exposure Prophylaxes* (PrEP)) OR (Pre Exposure Prophylaxis (PrEP)) OR (Prophylaxi, Pre-Exposure (PrEP)) OR (Prophylaxis, Pre-Exposure (PrEP))	#9(n=345)
	#2	("HIV"[Mesh]) OR (Human Immunodeficiency Virus) OR (Immunodeficiency Virus*, Human) OR (Virus*, Human Immunodeficiency) OR (Human Immunodeficiency Viruses) OR (Human T Cell Lymphotropic Virus Type III) OR (Human T-Cell Leukemia Virus Type III) OR (LAV-HTLV-III) OR (Lymphadenopathy-Associated Virus*) OR (Lymphadenopathy Associated Virus) OR (Virus*, Lymphadenopathy-Associated) OR (Human T Lymphotropic Virus Type III) OR (AIDS Virus*) OR (Virus*, AIDS) OR (Acquired Immunodeficiency Syndrome Virus) OR (HTLV-III)	
	#3	("Drug-Related Side Effects and Adverse Reactions"[Mesh]) OR (Side Effects of Drugs) OR (Drug Side Effect*) OR (Effects, Drug Side) OR (Side Effect*, Drug) OR (Adverse Drug Reaction*) OR (Drug Reaction*, Adverse) OR (Reactions, Adverse Drug) OR (Adverse Drug Event*) OR (Drug Event*, Adverse) OR (Drug Toxicity) OR (Toxicity*, Drug) OR (Drug Toxicities)	
	#4	#1 AND #2 AND #3	
	#5	"Creatinine"[Mesh] OR Krebiozen OR (Creatinine Sulfate Salt) OR (Salt, Creatinine Sulfate) OR (Sulfate Salt, Creatinine)	
	#6	#1 AND #2 AND #5	
	#7	"Liver Function Tests"[Mesh] OR (Function Test*, Liver) OR (Liver Function Test) OR (Test, Liver Function) OR (Tests, Liver Function)	
	#8	#1 AND #2 AND #7	
	#9	#4 OR #6 OR #8	
Central	#1	MeSH descriptor: [Pre-Exposure Prophylaxis] explode all trees	#9 (n=89)
	#2	MeSH descriptor: [HIV] explode all trees	
	#3	MeSH descriptor: [Drug-Related Side Effects and Adverse Reactions] explode all trees	
	#4	MeSH descriptor: [Creatinine] explode all trees	
	#5	MeSH descriptor: [Liver Function Tests] explode all trees	
	#6	# 1 AND #2 AND #3	
	#7	# 1 AND #2 AND #4	
	#8	# 1 AND #2 AND #5	
	#9	#6 OR #7 OR #8	
Embase	#1	'pre-exposure prophylaxis' OR 'pre exposure prophylaxis' OR 'pre-exposure prophylaxi' OR (prophylax*, AND 'pre exposure') OR ('pre exposure' AND prophylax* AND prep) OR (pre AND exposure AND prophylaxis AND prep) OR (prophylaxi, AND 'pre exposure' AND prep) OR (prophylaxis, AND 'pre exposure' AND prep)	#9 (n=134)
	#2	'human immunodeficiency virus infection'/exp OR ('hiv infections prevention':ti,ab,kw AND control:ti,ab,kw) OR 'human immunodeficiency virus encephalopathy'/exp	
	#3	'adverse drug reaction'/exp OR 'adverse drug effect':ti,ab,kw OR 'adverse drug event':ti,ab,kw OR 'adverse reaction, drug':ti,ab,kw OR 'drug adverse effect':ti,ab,kw OR 'drug adverse reaction':ti,ab,kw OR 'drug reaction,	

Database	Search	Strategies	Result
		adverse':ti,ab,kw OR 'drug side effect':ti,ab,kw OR ('drug-related side effects':ti,ab,kw AND 'adverse reactions':ti,ab,kw) OR 'long term adverse effects':ti,ab,kw OR ('metabolic side effects of drugs':ti,ab,kw AND substances:ti,ab,kw)	
	#4	#1 AND #2 AND #3	
	#5	'creatinine blood level'/exp OR ('Creatinine Sulfate Salt') OR ('Salt, Creatinine Sulfate') OR ('Sulfate Salt, Creatinine')	
	#6	#1 AND #2 AND #5	
	#7	'liver function test'/exp OR ('Function Test*, Liver') OR ('Liver Function Test') OR ('Test, Liver Function') OR ('Tests, Liver Function')	
	#8	#1 AND #2 AND #7	
	#9	#4 OR #6 OR #8	
LILACS	#1	MH:(profilaxia pré-exposição) OR (PrEP) OR (pre-exposure prophylaxis) OR (profilaxis pre-exposición) OR MH: D065129$	#4 (n=4)
	#2	MH: HIV OR VIH OR MH: D04.210.500.812.768$	
	#3	MH: Efeitos Colaterais e Reações Adversas Relacionados a Medicamentos) OR (Drug-Related Side Effects and Adverse Reactions) OR (Efectos Colaterales y Reacciones Adversas Relacionados con Medicamentos) OR (Adverse Drug Event*) OR (Adverse Drug Reaction*) OR (Adverse Event) OR (Drug Event*, Adverse) OR (Drug Reaction*, Adverse) OR (Drug Related Side Effects and Adverse Reactions) OR (Drug Side Effect*) OR (Drug Toxicity* Effects, Drug Side) OR (Reactions, Adverse Drug) OR (Side Effect, Drug*) OR (Side Effects, Drug) OR (Toxicity*, Drug) OR MH: D064420$	
	#4	#1 AND #2 AND #3	
ProQuest	#1	Pre-Exposure Prophylaxis" AND " HIV" AND " Adverse Reactions"	22
